# Supplementary material for: APOA1 Is a Novel Marker for Preeclampsia
Source: Int J Mol Sci. 2023 Nov 15;24(22):16363. doi: 10.3390/ijms242216363 (PMC10671820; doi:10.3390/ijms242216363)
Supplement: Supplementary file 1 [file ijms-24-16363-s001.zip › Supplementary Figure S1.pdf]

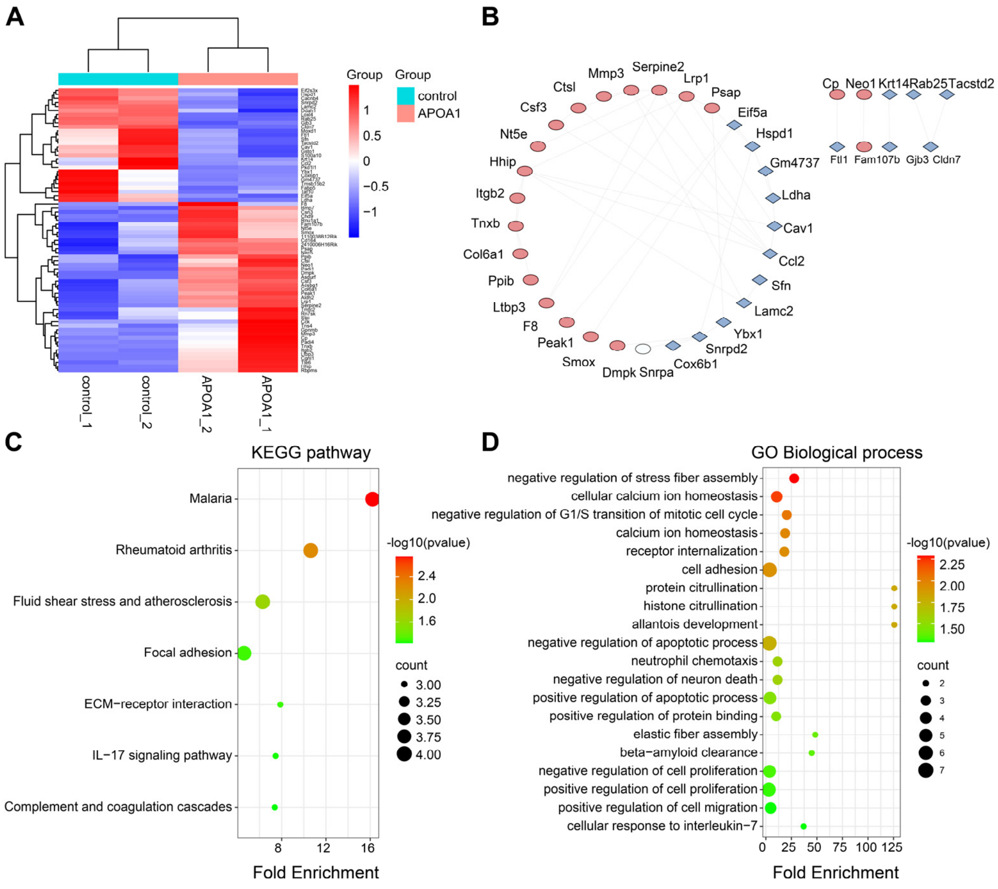

Figure S1 (A)The heatmap of the 70 DEGs. The ascending normalized expression level in the heatmaps was colored from blue to red. red means gene upregulation, blue indicates downregulation and white means normal expression. (B) The PPI network of 37 DEGs. red means gene upregulation, blue indicates downregulation and white means normal expression. (C-D) The enrichment map for the 70 DEGs generated using GO and KEGG pathway analyses, including all seven KEGG pathways (C) and top 20 GO biological process terms (D). Furthermore, the y axis represents the GO or KEGG terms, the x axis represents the enrichment score, the color stands for p-value, and the size of the circle represent the numbers of genes.
